# Recurrent Syncope Unveiling Pulmonary Hypertension Secondary to Pulmonary Artery Thrombi in a Pediatric Patient

**DOI:** 10.7759/cureus.51812

**Published:** 2024-01-07

**Authors:** Dina AlkhateebAltamimi, Karim Khalidi, Rima Khasawneh, Abdulhadi Alzaben, Khaled Salaymeh

**Affiliations:** 1 Pediatrics, Al Khalidi Hospital, Amman, JOR; 2 Interventional Radiology, Al Khalidi Hospital, Amman, JOR; 3 Pediatric Rheumatology, Yarmouk University, Amman, JOR; 4 Pediatric Oncology, Al Khalidi Hospital, Amman, JOR; 5 Pediatric Cardiology, Al Khalidi Hospital, Amman, JOR

**Keywords:** anti-phospholipid syndrome, anti-phospholipid antibodies, pulmonary emboli, pulmonary artery thrombosis, pulmonary hypertension

## Abstract

We present a case of a nine-year-old female patient who presented with recurrent syncope and was ultimately diagnosed with pulmonary hypertension (PH) secondary to pulmonary artery thrombi in the context of anti-phospholipid syndrome (APS). Extensive investigations including imaging studies revealed PH. Thromboembolic workup confirmed multiple pulmonary artery thrombi, and anti-phospholipid antibody testing confirmed APS. The patient received anticoagulation therapy tailored to APS management. Follow-up assessments demonstrated significant improvement in PH leading to cessation of syncope episodes. In this case, we underscore the importance of considering rare causes of syncope in the pediatric age group, particularly autoimmune disorders. Timely recognition and appropriate management are crucial for favorable outcomes in such cases. This report contributes to understanding the diverse clinical presentations of APS and emphasizes the need for a comprehensive diagnostic approach in patients with unexplained syncope.

## Introduction

Pulmonary hypertension (PH) in the pediatric age group stands as a clinical challenge, given its relatively low prevalence and diverse etiologies [1]. PH, characterized by elevated pulmonary artery pressure, carries significant morbidity and mortality, particularly when diagnosed in children [2]. This condition necessitates careful evaluation and management due to its potential impact on cardiac and pulmonary function. One intriguing facet of pediatric PH is its association with pulmonary thrombosis and emboli, a noteworthy contributor to the development and progression of the disease. Anti-phospholipid syndrome (APS), an autoimmune disorder characterized by vascular thrombosis among other manifestations, poses a particular challenge when coupled with pediatric PH. In the present case, we delve into the clinical presentation of a nine-year-old female patient who presented with recurrent syncope, leading to the discovery of severe PH in the context of APS confirmed by blood tests.

## Case presentation

A nine-year-old female patient presented to the pediatric cardiology outpatient clinic due to recurrent episodes of syncope. Her medical history dates back to nine months prior to presentation, when she experienced three episodes of syncope, all lasting for less than a minute and preceded by strenuous activity without other symptoms.

ECG revealed sinus rhythm with right ventricular hypertrophy. Echocardiography revealed severe pulmonary hypertension (PH) indicated by significantly dilated right atrium and right ventricle with fair right ventricular systolic function. Pulmonary artery systolic pressure was 60 mmHg. There was bowing of the interatrial septum from right to left. Left atrial and ventricular sizes were normal with normal left ventricular systolic function. No shunt lesion was identified.

**Figure 1:**
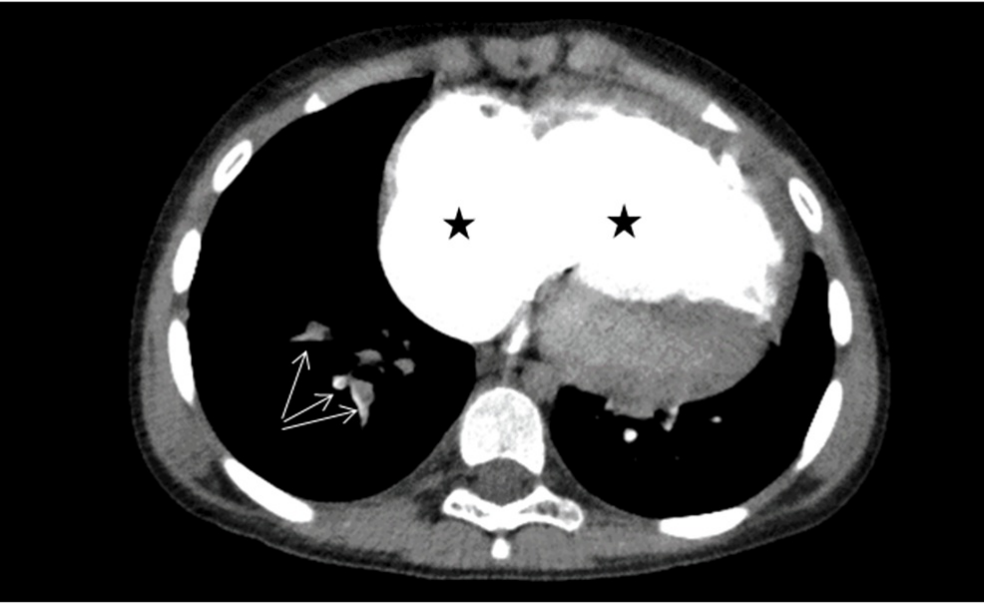
Chest CT angiography of the patient. Asterixis demonstrates enlargement of the right atrium and right ventricle secondary to right-sided heart strain because of pulmonary artery hypertension. Arrows point to filling defects (pulmonary emboli) in the segmental pulmonary arterial branches supplying the right lower lobe.

CT chest angiography showed extensive filling defects involving the pulmonary arterial branches of the right lower lobe in keeping with acute pulmonary emboli with features of chronic pulmonary emboli involving the remainder of the right and left lung lobes. There were additional imaging features of right-sided heart strain with enlargement of the right-sided heart chambers, flattening of the interventricular septum, enlargement of the main pulmonary trunk, and reflux of contrast into the inferior vena cava (IVC) (Figure 1).

Laboratory work-up was unremarkable for factor V Leiden, protein C, protein S, and anti-thrombin activity. However, it revealed positive anti-nuclear antibodies (ANA) (1:320) with negative double-stranded DNA, anti-Smith, and extractable nuclear antigens (ENAs). Testing for anti-phospholipid antibodies on two occasions, three months apart revealed persistently positive anti-cardiolipin and anti-beta 2 glycoprotein 1 antibodies and elevated lupus anticoagulant, thus fulfilling the 2006 European Alliance of Association for Rheumatology (EULAR) classification criteria for anti-phospholipid syndrome (Table 1). The retinal examination was normal without other system involvement.

**Table 1:** The patient’s anti-phospholipid laboratory work-up at diagnosis and follow-up.

Anti-phospholipid labs	At presentation	Three months later	Reference value
Lupus anticoagulant	31.4 s	49.6 s	<45 s
Anti-cardiolipin antibody - IgG	23.2 GPL	23.7 GPL	<10 GPL
Anti-cardiolipin antibody - IgM	2.3 GPL	3.1 GPL	<7 GPL
Anti-beta 2 glycoprotein 1 - IgG	59 GPL	53.1 GPL	<54 GPL
Anti-beta 2 glycoprotein 1 - IgM	2.3 U/mL	8.9 U/mL	<5 U/mL

A treatment plan was discussed with the parents with the patient being started on subcutaneous enoxaparin sodium injections 1 mg/kg/dose every 12 hours due to her high risk of thrombosis, along with oral phosphodiesterase-5 inhibitor (sildenafil) 12.5 mg three times daily for the pulmonary hypertension. The patient was advised to follow-up every three months. Unfortunately, the patient was lost on follow-up.

## Discussion

In this case, we present the clinical scenario of a female patient with PH caused by pulmonary artery thrombosis, and the subsequent identification of positive blood tests for APS. This case highlights the significance of recognizing the association between APS and PH, particularly in pediatric patients.

PH presents a complex and multifaceted clinical challenge that has garnered increasing attention within the pediatric medical community. It is a condition characterized by elevated pulmonary artery pressure >25 mmHg in children >3 months of age at sea level [1]. It is less common in children than in adults, with an estimated prevalence of <10 cases per 1 million children [3]. A classification system was developed by the Pulmonary Vascular Research Institute Pediatric Taskforce in Panama that states various causes of PH one of which is pediatric thromboembolic diseases [4]. Signs and symptoms of PH, when present, may include fatigue, dyspnea with exertion, and syncope that was present in our patient [5]. Many variables determine long-term survival with older age at diagnosis being significantly associated with decreased survival [6]. RV function and hypertrophy are important determinants of clinical status and outcomes prompting early performance of echocardiography [1]. Management of PH requires a multidisciplinary team. Targeted therapy with the phosphodiesterase-5 inhibitor sildenafil has been shown to decrease pulmonary arterial pressure and improve exercise capacity, and so was used as part of the treatment plan for our patient [7].

APS is an autoimmune disorder [8]. The European Alliance of Association for Rheumatology (EULAR) 2006 classification criteria are fulfilled when at least one clinical (vascular thrombosis and pregnancy morbidity) and one laboratory criteria (positive lupus anticoagulant or antiphospholipid antibodies) are present on ≥2 occasions at least 12 weeks apart. There is a high risk for thrombosis in patients who are positive for anti-cardiolipin, lupus anticoagulant, or those with multiple antibody positivity [9]. Pediatric APS is more common in females (male: female ratio 1.2-3:1) with a mean age of 10.7 years (range: 1-17.9 years) without reliable estimates for incidence and prevalence in children. About 50-60% of pediatric APS patients are of the secondary type (associated with autoimmune disorders), and about 20% of patients with primary APS eventually develop lupus [9]. The mainstay treatment of APS is anticoagulation, yet the optimal duration is not determined [10]. Despite treatment, there is a 3-24% risk of recurrence [10]. Patients should be followed up periodically through clinical evaluation and labs [9].

## Conclusions

Given the rarity of pediatric cases linking APS and PH, reporting such cases is crucial to enhance our understanding of this association. Early recognition and appropriate management can help improve patient outcomes and guide further research efforts in understanding this complex association.
